# Factor structure and longitudinal measurement invariance of the K6 among a national representative elder sample of China

**DOI:** 10.1186/s12889-022-14193-7

**Published:** 2022-09-21

**Authors:** Lisong Zhang, Zhongquan Li

**Affiliations:** 1 School of Sociology and Population, Nanjing University of Posts and Telecommunications, Nanjing, China; 2 School of Social and Behavioral Sciences, Nanjing University, 163 Xianlin Avenue, Qixia District, Nanjing, 210023 Jiangsu China

**Keywords:** Longitudinal measurement invariance, Psychological distress, The K6, Dimensionality, The elder

## Abstract

**Background:**

As the number of older people is rapidly growing, prevention, screening, and treatment of mental health problems (including anxiety and depression) in this population increasingly become a heavy burden to individuals, families, and even the whole society. The Kessler-6 screening measure (K6) is an efficient and effective instrument for general mental health problems. However, few studies have examined its measurement invariance across time, which is particularly important in longitudinal studies, such as exploring developmental trajectories of non-specific psychological distress and evaluating the effects of certain interventions.

**Methods:**

The current study investigated the factor structure and the longitudinal measurement invariance of the K6 among a national representative elder sample of China. Longitudinal data in two survey waves (the year 2010, and the year 2014) from the China Family Panel Studies were drawn for secondary data analysis. A total of 3845 participants aged 60 years old and above (52.2% male, mean age = 66.99 years, SD = 5.93 years) responded to both waves of the survey.

**Results:**

A comparison of four existing models with confirmatory factor analysis supported a two-factor solution of the K6. A series of multi-group confirmatory factor analyses further indicated that the K6 held strict longitudinal measurement invariance across time. Additionally, the internal consistency indices across time and the stability coefficients over time were acceptable.

**Conclusions:**

The findings further confirmed the psychometric defensibility of the K6 when used in the old Chinese population. The longitudinal measurement invariance justified comparisons of psychological distress scores among different measurement time points.

## Background

Mental-health problems, including anxiety and depression, are pretty common among the aging population. A report on National Mental Health Development in China (2017–2018) indicated that 11.51% to 22.02% were suffering from depression disorders among the Chinese older population, and 15% to 39.86% were struggling with anxiety disorders [1]. The China Health and Retirement Longitudinal Study (CHARLS) reported that the prevalence estimate of depression disorder was up to 33.09% [2]. As the number of older adults is rapidly growing, prevention, screening, and treatment of mental health problems in this population increasingly become a heavy burden to individuals, families, and even the whole society [3].

Several instruments have been developed or adapted for elderly populations to screen for general mental health problems, and The Kessler-6 screening measure (K6) is among these widely used ones [4]. It comprises six questions, which were drawn from the 10-item version of the Kessler Psychological Distress Scale for the purpose of screening severe mental illness among the general population fast and accurately [5, 6]. It may also be used in some clinical situations [7]. Moreover, due to effectiveness and efficiency, it is widely employed in major global and national surveys, such as the WHO World Mental Health (WMH) Survey, the US National Health Interview Survey [5], the Australian National Survey of Mental Health and Well-Being [8], the Canadian National Population Health Survey [9], the South African Stress and Health study [10], and the China Family Panel Studies [11].

However, researchers have not reached a consensus about the factor structure of the K6, which is vital in understanding and interpreting responses on this scale. The K6 was developed as a one-factor instrument at the beginning [6]. The one-factor model with all six items loading on a single factor is confirmed in the majority of studies [5, 8, 12–19]. Nevertheless, other factor solutions were proposed in a few studies, such as a modified single-factor model(with residual correlations among some items) [4], a two-factor model (with an item ("Everything was an effort") loading on the second factor) [5], a two-factor solution( with three items ("Nervous", "Restless or fidgety", and "Everything was an effort") on the anxiety factor, and another three items ("Hopeless", "Depressed", and "Worthless") on the depression factor) [20], a two-factor model (with four items formed the depression factor, and the rest two items ("Nervous" and "Restless or fidgety") formed the anxiety factor) and a second-order two-factor model [21, 22]. In a more recent study, we derived a two-factor model with exploratory factor analysis (EFA), with three items("Depressed", "Nervous", and "Restless or fidgety") loaded on the anxiety factor and the other three items("Hopeless", "Everything was an effort ", and "Worthless ") on the depression factor[3]. We also compared it with previous models using confirmatory factor analysis (CFA) and found that only this model was acceptable regarding model-data fit indexes. Therefore, in the present study, our first aim was to use a similar procedure to examine the dimensionality of the K6 in the elder sample with two waves of longitudinal survey data.

Measurement invariance (MI) refers to whether an instrument performs equivalently under different conditions [23]. Because researchers and practitioners often make comparisons on scores on instruments among different groups or settings, measurement invariance is considered an essential psychometric property of an instrument. Previous studies have examined measurement invariance of the K6 for gender, age, cultural groups and so on. Some confirmed measurement invariance of the K6 for various groups by conducting a series of multi-group confirmatory factor analyses [19, 24]. However, the others indicated measurement non-invariance between different groups [4, 9, 25]. In addition, some researchers attempted to address the measurement invariance issue with an item response theory approach. Sunderland et al. conducted differential item functioning analyses of the K6 with responses from Australian respondents aged between 16 and 85. They found significant item bias on one item (“Fatigue”) between the young and the old-aged groups [26].

The prior studies have focused on the measurement invariance of the K6 across different groups. However, to our knowledge, no study has examined the longitudinal measurement invariance (LMI) of the K6. That is, measurement invariance across different time points in the same sample [27]. The k6 is often used in longitudinal studies, and researchers want to know whether some changes emerge during the period or developmental trajectories of psychological distress [28]. If there is no guarantee of the longitudinal measurement invariance, the interpretation could also be misleading. Several scholars have realized the research gap in longitudinal measurement invariance of the K6, and call for future studies to address this issue [24]. Therefore, the second aim of the study was to check the degree to which the K6 demonstrates measurement invariance across time.

In sum, the present study was undertaken to examine the dimensionality of the K6 in a national representative elder sample in China and test the longitudinal measurement invariance of the K6 across time among this population.

## Methods

### Data and sample

This study was conducted based on second-hand data. The data came from two waves of the China Family Panel Studies (CFPS): the Year 2010 and the Year 2014. The CFPS was launched by the Institute of Social Science Survey of Peking University in the year 2010, funded by the Chinese government. It is a longitudinal survey conducted annually among Chinese national representative communities, families, and individuals. The survey covered various topics, from economic activities and education outcomes to family dynamics and relationships [11]. It employed multistage, implicit stratification, and probability proportion in sampling to obtain a nationally representative sample. Its baseline sample in the year 2010 wave covered 25 major provinces that represented 95% of the Chinese total population. According to further analysis of the sample, age and gender distributions were very similar to those of the 2010 6th National Population Census [29]. Both waves of the Year 2010 and the Year 2014 included the K6 as a measure of mental health. By pairing and deleting records with missing values, 3845 valid reaction data were finally retained. Among the final sample, there were 1836 females (47.8%) and 2009 males (52.2%). Their ages ranged from 60 to 110 years old (M = 66.99, SD = 5.93). The majority of them (55%) were from rural areas, with the rest (45%) from urban areas.

### Measures

#### The 6-item version of the Kessler psychological distress scale

The K6 is a brief version of the Kessler Psychological Distress Scale. It was developed from the 10-item version to measure psychological distress [5]. The Chinese version of the K6 has been validated in Chinese populations with Cronbach's alpha at 0.84, and the 32- to 53-day interval test–retest reliability at 0.79 [15]. The CFPS included the K6 in its survey of the years 2010 (time 1) and 2014 (time 2). Participants were asked to rate on a five-point Likert scale ranging from 1 (“All of the time”) to 5 (“None of the time”) on six items related to the following feelings during the past four weeks, such as sad, nervous, hopeless, and worthless. In the present analysis, the ratings for the individual item were recoded into a scale from 0(“None of the time”) to 4(“All of the time”) to align with prior studies. The sum scores of the six items were calculated as an index for psychological distress, with higher scores indicating more severe symptoms of anxiety and depression. The Cronbach alpha coefficient for the whole sample is 0.859 at time 1 and 0.871 at time 2, respectively.

### Statistical analyses

All the K6 items were rated on a five-point Likert scale. Firstly, we conducted descriptive statistics of the responses on the K6 with SPSS 26.0. Next, we conducted a series of confirmatory factor analyses to determine which model best fit the data with Mplus 7.4. Due to the highly skewed distribution of the response, we treated the data as categorical. The analysis employed the recommended polychoric correlation with weighted least squares with mean and variance adjusted (WLSMV) estimator [30]. Goodness-of-fit between model and data was assessed using the comparative fit index (CFI), the Tucker-Lewis index (TLI), and the root mean square error of approximation (RMSEA): CFI ≥ 0.90, TLI ≥ 0.90, RMSEA ≤ 0.08 [31, 32]. Finally, we tested the longitude measurement invariance of the K6 across time (The years 2010 and 2014) using a series of longitudinal confirmatory factor analyses. Following Little et al. [33], we included all measurement points in a model and allowed the residuals of corresponding items to covary across time points. For continuous data, researchers often use a set of four nested models to evaluate measurement invariance, i.e., configural, metric, scalar, and strict invariance. The configural invariance requires the same general measurement pattern of factor loading across different time points. The metric invariance further requires the identical factor loadings across time. The scalar invariance requires both invariant factor loadings and invariant intercepts across time. The strict factor invariance requires factor loadings, intercepts, and residual variances of items to be equal across occasions [34]. For categorical data, because the factor loadings and thresholds must be varied in tandem [35], the steps of longitudinal measurement invariance testing are a little different, and the metric invariance dropped from the procedure. Accordingly, a set of three nested models (configural invariance, strong invariance, and strict invariance) with increasing restrictive constraints were evaluated in the testing procedure for longitudinal measurement invariance with categorical data [34]. The summary of model specification is displayed in Table 1. As recommended by Cheung and Rensvold [36] as well as Chen [37], changes in CFI less than 0.01 and changes in RMSEA less than 0.015 between two consecutive models indicate that the more restrictive model can be considered equivalent to the less restricted model.

**Table 1:** Testing for longitudinal measurement invariance with categorical data(Model Specification)

	Factor loadings	Thresholds	Residual variances	Factor means
1 Configural invariance	(*	*)	Fixed at 1/1	Fixed at 0/0
2 Strong invariance	(Fixed	Fixed)	Fixed at 1/*	Fixed at 0/*
3. Strict invariance	(Fixed	Fixed)	Fixed at 1/1	Fixed at 0/*

The asterisk (*) indicates that the parameter is freely estimated across time; Fixed = the parameter is fixed to equity over time points; Fixed at 1/1 = residual variances are fixed at 1 at both time points; Fixed at 1/* = the residual variances are fixed at 1 at time 1 and freely estimated at time 2; Fixed at 0/0 = factor means parameters are fixed at 0 at both time points; Fixed at 0/* = factor means parameters are fixed at 0 at time 1 and freely estimated at time 2. Parameters in parentheses need to be varied in tandem

## Results

### Descriptive statistics

Table 2 shows the response distribution on five options for each item at both times. From the table, we can see that response distributions on each symptom are positively skewed. Most people endorsed the option “None of the time”, while only a few endorsed the option “Most of the time”. The prevalence rate of psychological distress is 4.5% at time 1 and 7.2% at time 2 in terms of the cut point of 12/13. Moreover, the standardized variance/covariance matrix (polychoric correlation) of items in two waves is displayed in Table 3.

**Table 2:** Responses distributed in five categories

	Year 2010					Year 2014				
Item	0	1	2	3	4	0	1	2	3	4
1.Depressed	2128(55.3%)	1230(32%)	164(4.3%)	120(6.0%)	93(2.4%)	2042(53.1%)	1157(30.1%)	274(7.1%)	253(6.6%)	119(3.1%)
2.Nervous	2472 (64.3%)	1055(27.4%)	107(2.8%)	170(4.4%)	41(1.1%)	2369(61.6%)	999(26.0%)	229(6.0%)	175(4.6%)	73(1.9%)
3.Restless or fidgety	2433(63.3%)	1023(26.6%)	153(4.0%)	175(4.6%)	61(1.6%)	2389(62.1%)	946(24.6%)	223(5.8%)	199(5.2%)	88(2.3%)
4.Hopeless	2839(73.8%)	684(17.8%)	114(3.0%)	158(4.1%)	50(1.3%)	2868(74.6%)	580(15.1%)	156(4.1%)	164(4.3%)	77(2.0%)
5.Everthing was an effort	2348(61.1%)	954(24.8%)	157(4.1%)	280(7.3%)	106(2.8%)	2275(59.2%)	862(22.4%)	219(5.7%)	303(7.9%)	186(4.8%)
6.Worthless	2901(75.4%)	674 (17.5%)	90(2.3%)	125(3.3%)	55(1.4%)	2852(74.2%)	593(15.4%)	160(4.2%)	158(4.1%)	82(2.1%)

*N* = 3845. 0 = None of the time, 1 = A little of the time, 2 = Some of the time, 3 = Most of the time, 4 = All of the time

**Table 3:** Standardized variance/covariance matrix (polychoric correlation)

	Item1_1	Item2_1	Item3_1	Item4_1	Item5_1	Item6_1	Item1_2	Item2_2	Item3_2	Item4_2	Item5_2	Item6_2
Item1_1	1.00											
Item2_1	0.70***	1.00										
Item3_1	0.70***	0.76***	1.00									
Item4_1	0.61***	0.58***	0.68***	1.00								
Item5_1	0.61***	0.61***	0.67***	0.72***	1.00							
Item6_1	0.61***	0.57***	0.64***	0.79***	0.72***	1.00						
Item1_2	0.26***	0.23***	0.26***	0.24***	0.27***	0.28***	1.00					
Item2_2	0.25***	0.28***	0.27***	0.21***	0.26***	0.25***	0.70***	1.00				
Item3_2	0.28***	0.29***	0.31***	0.26***	0.31***	0.28***	0.66***	0.78***	1.00			
Item4_2	0.25***	0.20***	0.26***	0.28***	0.27***	0.32***	0.63***	0.63***	0.68***	1.00		
Item5_2	0.29***	0.28***	0.30***	0.29***	0.37***	0.32***	0.59***	0.60***	0.67***	0.70***	1.00	
Item6_2	0.30***	0.26***	0.30***	0.34***	0.34***	0.38***	0.64***	0.64***	0.66***	0.79***	0.73***	1.00

***p < 0.001

### Examining factor structure

We conducted a series of confirmatory factor analyses to examine which one fit the data best among the four candidate models identified previously. These models included a one-factor model proposed by Kessler et al. with all items loaded on the same factor [6], a two-factor model proposed by Lee et al. with three items (Nervous", "Restless or fidgety", and "Everything was an effort") loaded on the anxiety factor and the rest three items ("Hopeless", "Depressed", and "Worthless") loaded on the depression factor [20], a two-factor model proposed Bessaha with two items ("Nervous" and "Restless or fidgety") loaded on the anxiety factor, while all the other four items on the depression factor [21], as well as our two-factor model, with three items("Depressed", "Nervous", and "Restless or fidgety") loaded on the anxiety factor, and the other three items("Hopeless", "Everything was an effort ", and "Worthless ") on the depression factor [3]. Table 4 shows the model goodness-of-fit indices. The fit indices indicated that our two-factor model was the only acceptable model for both time points (CFI and TLI > 0.90, RMSEA < 0.08). Therefore, this model served as a starting point for testing longitudinal measurement invariance.

**Table 4:** Model goodness-of-fit indices

Model	Year 2010					Year 2014				
	χ^2^	df	CFI	TLI	RMSEA(90%CI)	χ^2^	df	CFI	TLI	RMSEA(90%CI)
One-factor model(Kessler et al., 2002)	809.394	9	0.970	0.950	0.152(0.143, 0.161)	694.440	9	0.974	0.956	0.141(0.132, 0.150)
Two-factor model(Zhang & Li, 2020)	154.379	8	0.994	0.990	0.069(0.060, 0.079)	149.377	8	0.995	0.990	0.068(0.059, 0.078)
Two-factor model (Lee et al., 2012)	768.246	8	0.971	0.946	0.157(0.148, 0.167)	644.217	8	0.976	0.955	0.144(0.135, 0.153)
Two-factor model(Bessaha, 2015)	625.869	8	0.977	0.956	0.142(0.132, 0.151)	433.816	8	0.984	0.970	0.118(0.108, 0.127)

*N* = 3845. χ^2^, chi-square goodness of fit statistic; df, degrees of freedom; *CFI* Comparative fit index, *TLI* Tucker lewis index, *RMSEA* Root-mean-square error of approximation

### Longitudinal measurement invariance

The longitudinal measurement invariance model fit statistics for the K6 are displayed in Table 5. Firstly, we examined the configural invariance. In the model, all the factor loadings and thresholds are freely estimated without constraints for both time points, and the residual variances are fixed at 1 for identification purposes. The configural invariance model fit the data well (CFI = 0.995, TLI = 0.992, and RMSEA = 0.037). It indicated that the configural invariance of the K6 held over time. The K6 shares similar factor structures between the year 2010 survey and the year 2014 survey. Secondly, we examined the strong invariance. In the model, all factor loadings and thresholds are identical between both time points, and the residual variances are freely estimated without constraints. The strong invariance model fit the data well (CFI = 0.994, TLI = 0.994, and RMSEA = 0.033). No significant change in CFI, TLI, and RMSEA (ΔCFI = -0.001, ΔTLI = 0.002, Δ RMSEA = -0.004) indicated that strong invariance of the K6 held over time. Thirdly, we examined the residual variances. In the model, all factor loadings, thresholds, and residual variances are identical between both time points. The strict invariance model fit the data well (CFI = 0.993, TLI = 0.994, and RMSEA = 0.034). No significant change in CFI, TLI, and RMSEA (ΔCFI = -0.001, ΔTLI = 0, Δ RMSEA = 0.001) indicated that strict invariance of the K6 held over time. In sum, these results suggest that the two-factor solution of the K6 had longitudinal measurement invariance over four years. The standardized factor loadings for the longitudinal invariance model are shown in Table 6.

**Table 5:** Longitudinal measurement invariance model fit statistics for the K6

Model	χ ^2^	df	CFI	TLI	RMSEA(90%CI)	Δχ ^2^(Δdf)	ΔCFI	ΔTLI	ΔRMSEA
Configural	267.712	42	0.995	0.992	0.037 (0.033, 0.042)				
Strong	318.685	62	0.994	0.994	0.033 (0.029, 0.036)	65.352(20)	-0.001	0.002	-0.004
Strict	371.122	68	0.993	0.994	0.034 (0.031, 0.037)	55.475(6)	-0.001	0	0.001

*N* = 3845. χ^2^, chi-square goodness of fit statistic; df, degrees of freedom; *CFI* Comparative fit index, *TLI* Tucker lewis index, *RMSEA* Root-mean-square error of approximation; Δχ ^2^(Δdf), difference testing based on the DIFFTEST procedure for nested models with WLSMV; ΔCFI, change in Comparative Fit Index relative to the preceding model; ΔTLI, change in Tucker-Lewis Index relative to the preceding model; ΔRMSEA, change in Root-Mean-Square Error of Approximation relative to the preceding model

**Table 6:** Standardized factor loadings of the strict longitudinal invariance model for the K6

Item	Year 2010		Year 2014	
	**Anxiety**	**Depression**	**Anxiety**	**Depression**
1.Depressed	0.803		0.813	
2.Nervous	0.841		0.850	
3.Restless or fidgety	0.887		0.894	
4.Hopeless		0.867		0.878
5.Everything was an effort		0.837		0.849
6.Worthless		0.874		0.884

*N* = 3845

### Internal consistency, test–retest reliability, and stability coefficients across time

Regarding internal consistency, the coefficient for the K6 and its subscales were acceptable at both time points. The Cronbach alpha coefficient for the anxiety factor score is 0.803 at time1 and 0.809 at time 2. The Cronbach alpha coefficient for the depression factor score is 0.802 at time1 and 0.802 at time 2. The Cronbach alpha coefficient for the whole scale is 0.859 at time1 and 0.871 at time 2. Moreover, the test–retest reliability over four years is 0.265 for the first factor, 0.287 for the second factor, and 0.315 for the whole scale. Finally, we also computed the stability coefficients across time with the strict invariance model. That is a correlation between corresponding factors at both time points. The coefficient is 0.369 for the anxiety factor and 0.418 for the depression factor. In all, these findings indicate the stability of the K6 scores.

## Discussion

The K6 is a widely used instrument for measuring general mental health problems. However, the issue of its factor structure remains controversial though its factor structure has been explored in a variety of samples and situations. Moreover, few studies have examined the longitudinal measurement invariance across time. Therefore, the present study evaluated the factor structure of the K6 and also examined whether the same structure existed across time in a nationally representative sample of old Chinese people. The results confirmed a two-factor solution of the K6 and supported the cross-time measurement invariance of the K6.

Regarding the factor structure of the K6, there are different solutions in the literature: one-factor models and several two-factor models. Zhang and Li (2020) argued that the diverse findings might be due to the differences in samples and statistical methods. For example, some studies collected responses from the general population, and other studies investigated in more specific populations, such as adolescents and emerging adults [3]. Moreover, most studies explicitly or implicitly assumed the responses on the K6 as continuous. They used principal axis analysis or principal component analysis as the method to extract factors in the exploratory factor analysis or used a maximum likelihood estimator in the confirmatory factor analysis. However, in consideration of the highly skewed distribution of the responses on the K6, we treated the ratings as categorical and employed WLSMV, a recommended estimator for this kind of data, in the confirmatory factor analysis. In the present study, we also extended the exploration to a nationally representative sample of the old population in China. Moreover, we made comprehensive comparisons among available conceptual models. Our findings supported the two-factor model Zhang and Li (2020) proposed with data from surveys both in the years 2010 and 2014 [3]. In this model, three items are loading on the first factor, and the other times are loading on the second factor. The solution is slightly different from other factor structures regarding the item-factor belongings. “Depressed” was loaded with “Anxiety” and “Nervous”, and “Everything was an effort” was loaded with “Hopeless” and “Worthless”. The findings suggest that two subscale scores rather than one total score representing mental health states should be recommended for using the K6 among the elderly in China.

Measurement invariance is an important issue when we make comparisons among different groups or different points. The cross-sectional measurement invariance has been established across gender and age groups. However, previous studies haven’t addressed the issue of longitudinal measurement invariance. Longitudinal measurement invariance means the construct is equally measured across time for the same sample, ensuring that differences in observed scores over time reflect the fundamental changes in the latent construct measured by the instrument [38, 39]. The present study examined the longitudinal measurement invariance of the K6 in a nationally representative sample of the old Chinese population. We tested longitudinal measurement invariance at four different levels, configural, weak, and strict invariance. Results indicate that the two-factor structure holds strict longitudinal invariance across time, suggesting the K6 measures psychological distress at different time points. It also implies that differences in the K6 scores should be considered true changes in a person's mental health. These findings justify the use of the K6 in studies for developmental or interventional purposes.

The present study contributes to current literature on exploring the psychometric properties of the K6 in at least two important ways. First, in contrast to most previous studies, we focused on an old population from Eastern cultures. And the sample has a good representation of Chinese elders, and the size is relatively large. Second, to our knowledge, we are among the first to check whether the K6 holds longitudinal measurement invariance over time. The study also has some limitations. First, we only have two waves of data in the analysis, and the interval of the waves is four years. Data of more waves and more diverse intervals are needed to replicate the finding in the present study. We only examined the factor structure and longitudinal measurement invariance in the general aged population. The testing should be extended to more specific aged populations or populations at other stages in life. In addition, the data was collected in the years 2010 and 2014, and it is relatively far from now. The results may be more valuable if more recent data are available for testing.

## Conclusions

In general, our study contributes to the literature on the K6 by expanding the investigation of its factor structure and longitudinal properties. The K6 holds strict longitudinal measurement among a nationally representative elder sample of China, which is of particular importance when used to examine the effects of some interventions, developmental trajectories of psychological distress, and other cases in longitudinal studies with elder samples.

## Data Availability

The raw data and the Mplus syntax is publicly available at https://osf.io/rp9x7/?view_only=c7bd63116e54408e91228124b388e228 If one needs more information, please contact the corresponding author, Prof. Zhongquan Li.

## Abbreviations

K6: The 6-item version of the kessler psychological distress scale
EFA: Exploratory factor analysis
CFA: Confirmatory factor analysis
MI: Measurement invariance
LMI: Longitudinal measurement invariance
CFPS: The china family panel studies
WLSMV: Weighted least squares with mean and variance adjusted
CFI: Comparative fit index
TLI: Tucker lewis index
RMSEA: Root-mean-square error of approximation
